# Immune activation and chronic inflammation: Is there an additional effect of HIV in a geriatric population?

**DOI:** 10.1097/MD.0000000000025678

**Published:** 2021-04-30

**Authors:** Delphine Sauce, Valérie Pourcher, Tristan Ferry, Jacques Boddaert, Laurence Slama, Clotilde Allavena

**Affiliations:** a Sorbonne Université, INSERM, Centre d’Immunologie et des Maladies Infectieuses (CIMI-Paris); b Assistance Publique-Hôpitaux de Paris, Sorbonne Université, Department of Infectious Diseases, Pitie-Salpetriere Hospital, Pierre Louis Institute of Epidemiology and Public Health, Sorbonne Université, Paris; c Hospices Civils de Lyon, Croix-Rousse Hospital, Department of Infectious Diseases, Centre International de Recherche en Infectiologie, CIRILyon; d Geriatrics department, Pitie-Salpetriere Hospital, UPMC; e Assistance Publique-Hôpitaux de Paris, Department of Infectious Diseases Hotel Dieu Hospital, Paris; f Department of Infectious Diseases, Hôtel Dieu CHU Nantes, INSERM UIC 1413, CHU Nantes, France.

**Keywords:** comorbidities, geriatric population, HIV population, immune activation, inflammation

## Abstract

HIV infection has become a chronic disease, with a lower mortality, but a consequent increase in age-related noninfectious comorbidities. Metabolic disorders have been linked to the effect of cART as well to the effects of immune activation and chronic inflammation. Whereas it is known that aging is intrinsically associated with hyperinflammation and immune system deterioration, the relative impact of chronic HIV infection on such inflammatory and immune activation has not yet been studied focusing on an elderly HIV-infected population.

The objectives of the study were to assess 29 blood markers of immune activation and inflammation using an ultrasensitive technique, in HIV-infected patients aged ≥75 years with no or 1 comorbidity (among hypertension, renal disease, neoplasia, diabetes mellitus, cardiovascular disease, stroke, dyslipidemia, and osteoporosis), in comparison with age-adjusted HIV-uninfected individuals to identify whether biomarkers were associated with comorbidities. Wilcoxon nonparametric tests were used to compare the levels of each marker between control and HIV groups; logistic regression to identify biomarkers associated to comorbidity in the HIV group and principal component analysis (PCA) to determine clusters associated with a group or a specific comorbidity.

A total of 111 HIV-infected subjects were included from the Dat’AIDS cohort and compared to 63 HIV-uninfected controls. In the HIV-infected group, 4 biomarkers were associated with the risk of developing a comorbidity: monocyte chemoattractant protein-1 (MCP-1), neurofilament light chain (NF-L), neopterin, and soluble CD14. Six biomarkers (interleukin [IL]-1B, IL-7, IL-18, neopterin, sCD14, and fatty acid-binding protein) were significantly higher in the HIV-infected group compared to the control group, 11 biomarkers (myeloperoxydase, interleukin-1 receptor antagonist, tumor necrosis factor receptor 1, interferon-gamma, MCP-1, tumor necrosis factor receptor 2, IL-22, ultra sensitivity C-reactive protein, fibrinogen, IL-6, and NF-L) were lower. Despite those differences, PCA to determine clusters associated with a group or a specific comorbidity did not reveal clustering nor between healthy control and HIV-infected patients neither between the presence of comorbidity within HIV-infected group.

In this highly selected geriatric HIV population, HIV infection does not seem to have an additional impact on age-related inflammation and immune disorder. Close monitoring could have led to optimize prevention and treatment of comorbidities, and have limited both immune activation and inflammation in the aging HIV population.

## 1 Introduction

Since the availability of potent and well tolerated combined antiretroviral treatment (cART), HIV infection has become a chronic disease, with a lower mortality, but a consequent increase in age-related noninfectious comorbidities. HIV-infected patients experience accentuated aging and multiple morbidities, but are most often disconnected from geriatric care. Several studies confirmed the highest prevalence of comorbidities in the HIV-infected aging population compared to the general population.^[1–5]^ Metabolic disorders have been linked to a prolonged exposition to first generation of cART as well as to the effects of immune activation and chronic inflammation. In the geriatric population where aging is *per se* associated with hyperinflammation and immune impairment,^[6]^ the relative burden of HIV infection on inflammatory and immune disorders has not been studied to date in this specific population. The objectives of this study were to identify inflammatory biomarkers which could explain the immune disturbances in an elderly HIV-infected population with no more than 1 comorbidity (≤1), and to compare them with healthy HIV-uninfected individuals to improve the clinical management and evaluate new therapeutic strategies, while HIV-infected patients are aging.^[7]^

## 2 Patients and methods

Dat’AIDS cohort is a collaboration of 22 major French HIV centers (NCT 02898987 clinicaltrials.gov) validated by an ethical committee (2017-A00928–45). The data collection has been accepted by the French national commission on informatics and liberty and the present study has been approved by the ethic committee.

All HIV-infected patients aged ≥75 years were selected with a regular follow-up confirming the clinical status at censoring date (January 31, 2016) and included if they had 0 to 1 comorbidity among: hypertension, renal disease (confirmed MDRD <60 mL/mn), neoplasia, diabetes mellitus, cardiovascular disease, stroke, dyslipidemia, and osteoporosis.

The HIV-infected group was compared to an age-matched healthy HIV-uninfected group (ie, control group). Elderly participants were included within the geriatrics department of Pitié-Salpétrière hospital and were selected according to rigorous inclusion criteria: absence of previous physical disabilities, absence of cognitive disorders that could interfere with the signing of the consent form and free of medication and of diseases affecting the immune system (ie, cancers, advanced chronic diseases such as uncontrolled cardiovascular disease, diabetes mellitus, chronic obstructive pulmonary disease). Subjects with acute clinical events (<3 months) and subjects treated with immunosuppressive therapy were excluded.

The objectives were to assess blood markers of immune activation and inflammation from cryopreserved sera, using ultrasensitive techniques (Luminex & Simoa) to identify whether biomarkers were associated with comorbidities. The 29 inflammatory biomarkers were selected based on their availability and relevance found in previous studies^[8–10]^ demonstrating pathophysiological relation between levels of these cytokines/chemokines and the development of comorbidities. Among the cytokines: interleukin (IL)-1β, which causes a number of different auto-inflammatory syndromes; IL-1RA, which is a member of the IL-1 family that binds to IL-1 receptors, but does not induce any intracellular response; IL-6, which could act as a pro- and anti-inflammatory cytokine; IL-12, which is secreted mostly by B cells upon bacteria challenges; IL-18, which is produced by macrophages; IL-7, which is a cytokine derived from T cells that enhances T cell homeostasis; IL-4, a cytokine that stimulates the proliferation of activated B-cells; IL-17A and IL-22, which are proinflammatory cytokines produced by helper T-cells, and act in concert with tumor necrosis factor-alpha (TNF-α) and IL-1 to promote cytokines production (IL-6, tumor growth factor-beta [TGF-β], IL-8) and therefore are potent mediators of cellular inflammatory responses. Other mediators such as Interferon-gamma (IFN-γ), TNF-α, and neopterin are also critical for innate and adaptive immunity. Receptors such as tumor necrosis factor receptor 1 (TNFR1) and tumor necrosis factor receptor 2 (TNFR2) are essential to modulate apoptosis and inflammation. Myeloperoxidase (MPO) is an enzyme most abundantly present in neutrophil granulocytes; it uses hydrogen peroxidase to convert chloride to hypochlorous acid which may destroy bacteria. In many inflammatory pathologies, this leads to tissue damage. We also measured 2 anti-inflammatory molecules, IL-10, which inhibits the synthesis of IFN-γ and TNF-α and latency-associated peptide of transforming growth factor beta 1 (LAP), which is an indirect sign of TGF-β secretion by macrophages, involved in cancer, heart disease, and immunity. We also included three chemokines: monocyte chemoattractant protein-1 (MCP-1), IL-8, and interferon-inducible protein 10 (IP-10), the latter of which induces the production of IFN-γ. The plasma level of intestinal-type fatty acid-binding protein (iFABP) reflects epithelial-cell layer apoptosis, which promotes microbial translocation followed by soluble CD14 (sCD14) as a marker of detection of bacterial lipopolysaccharide.

In addition, we used high sensitivity C-reactive protein (usCRP) and serum amyloid A (SAA), which are acute phase proteins associated with main inflammatory responses from tissue injury, trauma, infections, and neoplastic diseases.

Troponin and d-dimer are important biomarkers for noninvasive detection of myocardial injury in many cardiovascular events (coronary heart disease, thrombosis, and so on). Fibrinogen is a plasma glycoprotein that is the final common reaction of the coagulation cascade after thrombin cleaves soluble fibrinogen to form fibrin. Low levels of fibrinogen are seen in association with fibrinolysis and liver disease. A high level of fibrinogen is a risk factor for thrombosis and is a strong predictor of cardiovascular risk and stroke. Neurofilament light chain (NF-L) and Tau are biomarkers of neurological disorders and neurodegenerative diseases, respectively.

Measurements of the biomarkers were performed in a centralized laboratory (INSERM U1135, Paris).

Plasmatic levels of fibrinogen, usCRP, d-dimers, SAA, MPO, IP-10, MCP-1, IL-1Ra, IL-1b, IL-4, IL-7, IL-8, IL-10, IL-12p70, IL-17A, IL-18, IL-22, IFN-γ, TNF-α, TNFR1, TNFR2, and LAP were measured by Procartaplex kits (Thermofisher). Neopterin concentration, as well as iFABP2 and sCD14 were followed by Elisa (Tecan, Lyon, France; R&D systems, Abington, UK, respectively), whereas we used digital ultrasensitive Elisa to assess the concentration of IL-6, troponin, Tau, NF-L (Simoa technology, Quanterix, Lexington, MA). All measurements were performed according to manufacturer's recommendations.

### 2.1 Statistical analyses

Patients’ characteristics were described using median and interquartile (IQR) for continuous variables and sample size and percentage for categorical variables. HIV-infected patients and controls were compared using the nonparametric Kruskal-Wallis or Wilcoxon tests. In HIV-infected patients, a series of univariate logistic regression were fitted to identify which inflammatory biomarkers were prognostic factors of the presence of 1 comorbidity. All biomarkers leading to a *P* value <.20 were potentially selected in the final multivariate model using a stepwise procedure.

Variables were standardized by subtracting their mean and dividing by their standard deviation. Thereafter, we performed a principal component analysis (PCA) on the 29 biomarkers with the objective to identify, in HIV-infected patients, clusters of patients with comorbidity (0 vs 1 comorbidity) or type of comorbidity (cardiovascular vs diabetes vs HTA vs neoplasia vs other). Analyses were done by a statistician with SAS 9.4 and the R function PCA.

## 3 Results

A total of 111 HIV-infected subjects were included from the Dat’AIDS cohort of whom 39 without comorbidity with a median age of 81 years (IQR 78–84) and a median duration of HIV infection and cART of 18.2 (12–23) and 15.9 (9–19) years, respectively. They were compared to a control group of 63 individuals (median age of 83.4 years [IQR 78–89]). Subjects’ characteristics are shown in Table 1.

**Table 1:** Cohorts characteristics.

N (%) or median (IQR)	HIV group		Control group	
N	111		63	
Age, y	81	(78–84)	83.4	(79.7–88.8)
Male	80	(72.1)	20	(31.7)
French-native	88	(79.3)	—	
Duration of HIV infection, y	18.2	(11.8–22.5)	—	
Hepatitis HCV/HBV coinfection	7	(6.3)	—	
CDC stage C	29	(26.1)	—	
Tobacco active smokers	4	(3.6)	—	
Body mass index, kg/m^2^	23.4	(21.6–25.8)	—	
On ART	110	(99.1)	—	
HIV RNA <50 copies/mL	104	(94.6)	—	
Duration of HIV control, mo	100	(37–142)	—	
Nadir CD4, cells/mm^3^	174	(79.2–260)	—	
Current CD4, cells/mm^3^	510	(353–680)	585	(401–777)
CD4:CD8 ratio	0.64	(0.4–1.1)	2.3	(1.6–3.3)
IgG CMV-positive	74	(66.7)	50.8	(69.6)
No comorbidity	39	(35.1)	7	(11.1)
1 Comorbidity	72	(64.9)	23	(36.5)
≥2 Comorbidities		-	33	(52.4)
Diabetes mellitus	14	(12.6)	6	(9.5)
Hypertension	23	(20.7)	23	(36.5)
Cardiovascular diseases	7	(6.3)	8	(12.7)
Stroke	4	(3.6)	7	(11.1)
Dyslipidemia	1	(0.9)	—	
Kidney disease	1	(0.9)	45	(71.4)
Osteoporosis	1	(0.9)	—	
Neoplasia	21	(18.9)	9	(14.3)

Six biomarkers (IL-1β, IL-7, IL-18, neopterin, sCD14, and iFABP2) were significantly higher in the HIV group compared to the control group (Fig. 1  A), whereas 11 biomarkers (MPO, MCP-1, IFN-γ, TNFR1, TNFR2, IL-1RA, IL-22, IL-6, usCRP, NF-L, and fibrinogen) were lower (Fig. 1  B).

**Figure 1:**
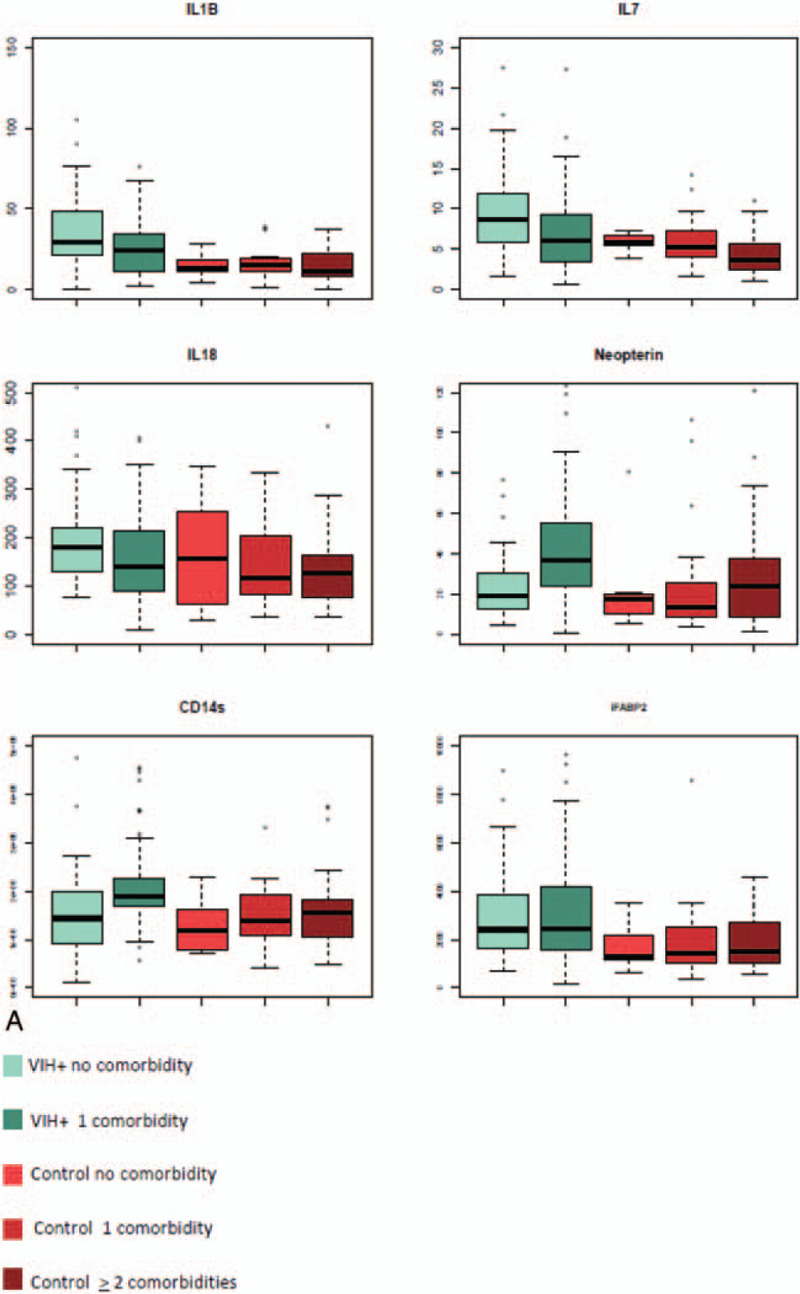
(A) Plasmatic level of inflammatory molecules: Distribution of elevated biomarkers in the HIV-infected group (with 1 [n = 72] or without comorbidity [n = 39] compared to the control group without [n = 7], with 1 [n = 23], and with >1 comorbidity [n = 33]). (B) Plasmatic level of inflammatory molecules: distribution of decreased biomarkers in the HIV-infected group (with 1 [n = 72] or without comorbidity [n = 39] compared to the control group (without [n = 7], with 1 [n = 23] and with >1 comorbidity [n = 33]). (C) Plasmatic level of inflammatory molecules: Distribution of biomarkers for which the level is equivalent in HIV-infected patients compared to healthy elderly controls. (D) Scatter plot of a 2 components of principal component analysis (PCA) identifying the degree of variance of the predicted variable (infection) explained by the 29 biomarkers measured and tested variables in regularized logistic regression. (E) PCA on the 29 biomarkers carried out in HIV-infected patients to identify clusters of patients with comorbidity or type of comorbidity. iFABP2 = intestinal fatty acid binding protein 2, IFN-γ = interferon Gamma, IL-1b = interleukin-1beta, IL-1RA = interleukin-1 receptor antagonist, LAP = latency associate peptide, MCP-1 = monocyte chemoattractant protein-1, MPO = myeloperoxydase, NF-L = neurofilament light chain, SAA = serum amyloid A, sCD14 = soluble CD14, TNFR1 = tumor necrosis factor receptor 1, TNFR2 = tumor necrosis factor receptor 2, usCRP = ultra sensitivity C-reactive protein.

**Figure 1 (Continued):**
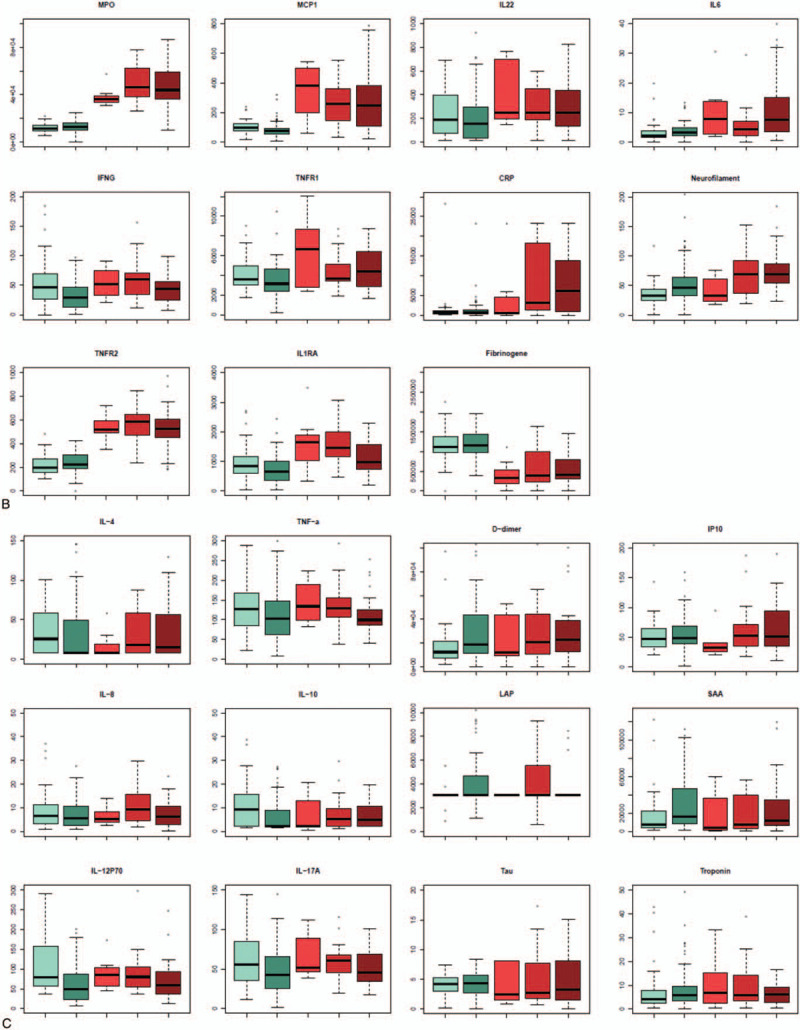
(A) Plasmatic level of inflammatory molecules: Distribution of elevated biomarkers in the HIV-infected group (with 1 [n = 72] or without comorbidity [n = 39] compared to the control group without [n = 7], with 1 [n = 23], and with >1 comorbidity [n = 33]). (B) Plasmatic level of inflammatory molecules: distribution of decreased biomarkers in the HIV-infected group (with 1 [n = 72] or without comorbidity [n = 39] compared to the control group (without [n = 7], with 1 [n = 23] and with >1 comorbidity [n = 33]). (C) Plasmatic level of inflammatory molecules: Distribution of biomarkers for which the level is equivalent in HIV-infected patients compared to healthy elderly controls. (D) Scatter plot of a 2 components of principal component analysis (PCA) identifying the degree of variance of the predicted variable (infection) explained by the 29 biomarkers measured and tested variables in regularized logistic regression. (E) PCA on the 29 biomarkers carried out in HIV-infected patients to identify clusters of patients with comorbidity or type of comorbidity. iFABP2 = intestinal fatty acid binding protein 2, IFN-γ = interferon Gamma, IL-1b = interleukin-1beta, IL-1RA = interleukin-1 receptor antagonist, LAP = latency associate peptide, MCP-1 = monocyte chemoattractant protein-1, MPO = myeloperoxydase, NF-L = neurofilament light chain, SAA = serum amyloid A, sCD14 = soluble CD14, TNFR1 = tumor necrosis factor receptor 1, TNFR2 = tumor necrosis factor receptor 2, usCRP = ultra sensitivity C-reactive protein.

**Figure 1 (Continued):**
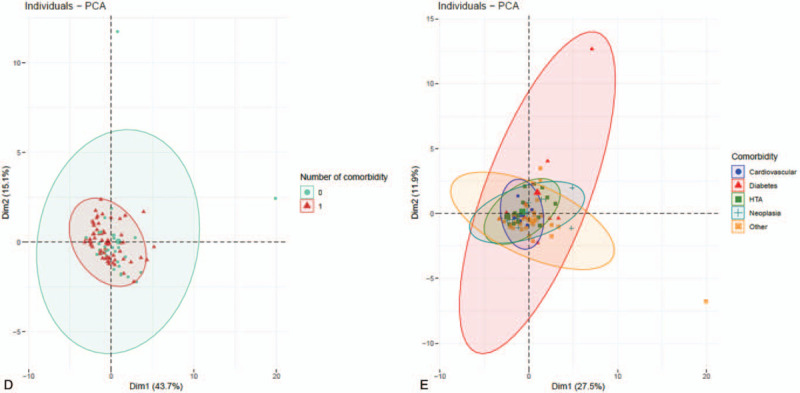
(A) Plasmatic level of inflammatory molecules: Distribution of elevated biomarkers in the HIV-infected group (with 1 [n = 72] or without comorbidity [n = 39] compared to the control group without [n = 7], with 1 [n = 23], and with >1 comorbidity [n = 33]). (B) Plasmatic level of inflammatory molecules: distribution of decreased biomarkers in the HIV-infected group (with 1 [n = 72] or without comorbidity [n = 39] compared to the control group (without [n = 7], with 1 [n = 23] and with >1 comorbidity [n = 33]). (C) Plasmatic level of inflammatory molecules: Distribution of biomarkers for which the level is equivalent in HIV-infected patients compared to healthy elderly controls. (D) Scatter plot of a 2 components of principal component analysis (PCA) identifying the degree of variance of the predicted variable (infection) explained by the 29 biomarkers measured and tested variables in regularized logistic regression. (E) PCA on the 29 biomarkers carried out in HIV-infected patients to identify clusters of patients with comorbidity or type of comorbidity. iFABP2 = intestinal fatty acid binding protein 2, IFN-γ = interferon Gamma, IL-1b = interleukin-1beta, IL-1RA = interleukin-1 receptor antagonist, LAP = latency associate peptide, MCP-1 = monocyte chemoattractant protein-1, MPO = myeloperoxydase, NF-L = neurofilament light chain, SAA = serum amyloid A, sCD14 = soluble CD14, TNFR1 = tumor necrosis factor receptor 1, TNFR2 = tumor necrosis factor receptor 2, usCRP = ultra sensitivity C-reactive protein.

Despite those differences, PCA analysis did not reveal clustering between healthy controls and HIV-infected patients, probably due to the fact that another set of 12 molecules did not reveal differences between the different groups of subjects (Fig. 1  C and D).

In the HIV group, 4 biomarkers were associated with the risk of comorbidity: MCP-1 (odds ratio [OR] 0.78, 95% CI 0.68–0.91, NF-L (OR 1.42, 95% CI 1.08–1.87), Neopterin (OR 1.99, 95% CI 1.33–2.97), and sCD14 (OR 1.01, 95% CI 1.00–1.02) (Fig. 1  E).

However, PCA did not discriminate between the absence or the presence of comorbidity within HIV group.

## 4 Discussion

In our study, no specific inflammatory or immune profile has been identified in HIV-infected geriatric patients compared to HIV noninfected geriatric population. Similarly, within HIV group, no signature was found to be associated with specific comorbidity signature. This can be explained by the fact that HIV-infected patients included in our study were highly selected: indeed, <4% of HIV-infected subjects are aged 75 years and older in the French HIV population. Moreover, we focused on patients who exhibit only 1 comorbidity despite their advanced age. Therefore, our HIV group could be considered as “survivors” with a very favorable phenotype. One indirect proof of this “healthy” status is based on the observation that this geriatric population achieve similar high rate of HIV virologic suppression than younger HIV-infected population (50–74 years).^[11]^ Recently, it has been described that elderly HIV-infected patients with few comorbidities had a better hemostatic profile (based on antithrombin and prothrombin time activities) than elderly HIV-uninfected control group, reflecting the success of treatment. As suggested by the authors, this better hemostatic balance in HIV-infected patients may be related to controlled disease status and better personal care compared to uninfected individuals.^[12]^

To date, few data were published concerning inflammatory and immune activation biomediators and the impact of aging in HIV infection.^[13–15]^ Substantial evidence suggest that chronic inflammation and immune activation contribute significantly to chronic conditions in people aging with or without HIV infection. Of interest, our data showed a sustained higher level of sCD14 in our group of HIV-infected elderly patients, suggesting a continuous low-grade immune activation related to gut leakage. Similarly to a recent study, people living with HIV had significantly higher levels of sCD14 in plasma as compared to HIV noninfected subjects despite differences between the HIV groups evaluated in those 2 studies mainly on age and ART history.^[16]^ Contradictory results have been reported from different studies on the effect of cART on sCD14.^[17–20]^ These data suggest that HIV-infected individuals, even those on long-term successful cART, may be at higher risk of developing inflammatory diseases leading to inflammaging. The low-grade chronic inflammation is a major risk factor for the development of many age-associated diseases and to some extent morbidity and mortality in elderly people.

Moreover, in vivo relationships between serum levels of neopterin, a biomarker of immune activation, and 4 commonly described inflammatory biomediators: TNFR1, TNFR2, IL-6, and CRP as well as the impact of HIV infection and aging have been previously evaluated in drug users with or at high risk for HIV infection.^[21]^ Multivariate regression, performed in this study, showed significant impact of age-associated comorbidities on all 5 biomediators and that of HIV infection on all but TNFR1. In the adjusted model, neopterin had positive associations with TNFR1 and TNFR2. These findings may also facilitate elucidation of underlying inflammatory and immune activation pathways that contribute to age-related chronic conditions, potentially leading to identification of key biomediators, particularly those upstream of CRP, as novel targets for intervention. Consistent with the data reported in the general geriatrics population, recent data from 2 large cohort studies of HIV-infected participants, the Multicenter AIDS Cohort Study (MACS) in men who have sex with men and the AIDS Linked to the Intravenous Experience study in people with a history of injection drug use, have reported evidence for a role of immunosenescence, particularly chronic low-grade inflammatory phenotype in the pathogenesis of frailty.^[22,23]^ Results from the MACS demonstrated an association of frailty with higher concentrations of inflammatory markers as well as with higher concentrations of IL-6 and CRP and greater proportions of lymphocytes with the senescent T cell phenotype.^[24]^ Activation of monocytes and macrophages, marked by increased levels of neopterin, may contribute to chronic inflammation in the frail older population.^[25,26]^ In our study, neopterin was significantly higher in HIV group and associated with the risk of comorbidity, suggesting that older HIV-infected individuals could be more frail than uninfected elderly. However, CRP and IL-6 levels in our HIV-infected patients did not increase compared to uninfected controls. The absence of difference in our 2 geriatric populations highlights probably a better follow-up in terms of daily screening and treatment of comorbidities.

Our study has some strength and limitation; the number of included subjects is low but is due to the highly selected elderly HIV infected population and some comorbidities could have been underdiagnosed or under-reported. However, the evaluation of some markers using an ultrasensitive assay reinforces our results. As our study aims to evaluate the impact of HIV on immune activation and inflammation in an elderly population, a highly selected HIV-infected and HIV-uninfected population was included. That does not allow us to generalize our results to the elderly population with numerous age-associated comorbidities. With regard to sex, the 2 groups are different because of a bias related to HIV infection. Male are still over-represented in the cohorts of HIV-infected participants in Northern countries and this sex ratio increases in aging population. However, the over-representation of female increases with age in the general population. However, several recent articles show that some markers vary by sex, but not always in the same way.^[27]^ This issue should be taken into account but still debated. In our study it should be noted that no association was seen after adjustment. Another difference between the 2 groups is the highest proportion of renal disease in the control group. This difference is mainly explained by the age difference between the 2 groups. If we take into account age by dividing patients according to the median age (81 years), the difference disappears in each age group (data not shown).

Similar to the present need for primary care providers to manage chronic noninfectious comorbidities among elderly persons with well-controlled HIV infection, HIV clinical care will need to routinely involve geriatric medicine in a new HIV-geriatric discipline. In general population in France, no recommendation exists according to the optimal follow-up of geriatric population. In Italy, guidelines have suggested the screen for frailty in people living with HIV aged >50 years.^[28]^ In the last European EACS recommendation, new data have been added on the prescription of antiretroviral drugs in elderly and in the recognition of frailty.^[29]^ More broadly, American guidelines underline that HIV experts, primary care providers, and other specialists should work together to optimize the medical care of older patients with HIV and complex comorbidities.^[30]^ Screening for geriatric syndromes is both a multidisciplinary and multidimensional process, designed to evaluate an older person's functional ability, physical health, cognition, overall mental health, and socioenvironmental circumstances. With the aging of HIV-infected population, routine incorporation of geriatric assessment into clinical trials involving HIV-infected patients should be encouraged. This awareness is however dampened due to the shortage of geriatrics experts and the absence of consensus and tests defining frailty and vulnerability worldwide.

## 5 Conclusion

As a result of successful antiviral therapy, HIV-infected geriatric patients are now living longer, leading to an emerging vulnerable population, and the question is how to best care for them. In this study based on highly selected geriatric HIV population, HIV infection does not seem to have an additional impact on age-related inflammation and immune disorder, suggesting that HIV-patients are well taken in care and followed. It is therefore encouraging to observe that prevention and treatment of comorbidities could have limited both immune activation and inflammation in the aging HIV population. To validate this preliminary hypothesis, other studies need to be performed on a large cohort of geriatric HIV-infected patients.

## Acknowledgments

The authors thank Philippe Flandre (IPLESP INSERM 1146, Paris France) and Solène Sécher (CHU Nantes, France) for the statistical analysis.

## Author contributions

**Conceptualization:** Delphine Sauce, Valérie Pourcher, Tristan Ferry, Laurence Slama, Clotilde Allavena.

**Formal analysis:** Delphine Sauce.

**Funding acquisition:** Delphine Sauce, Clotilde Allavena.

**Investigation:** Delphine Sauce, Valérie Pourcher, Clotilde Allavena.

**Methodology:** Delphine Sauce, Valérie Pourcher, Tristan Ferry, Jacques Boddaert, Laurence Slama, Clotilde Allavena.

**Project administration:** Clotilde Allavena.

**Resources:** Delphine Sauce.

**Supervision:** Delphine Sauce, Clotilde Allavena.

**Validation:** Delphine Sauce, Valérie Pourcher, Clotilde Allavena.

**Visualization:** Delphine Sauce, Valérie Pourcher, Clotilde Allavena.

**Writing – original draft:** Delphine Sauce, Valérie Pourcher, Clotilde Allavena.

**Writing – review & editing:** Delphine Sauce, Valérie Pourcher, Tristan Ferry, Jacques Boddaert, Laurence Slama, Clotilde Allavena.
